# Inflammatory Markers in Older Women with a History of Gestational Diabetes and the Effects of Weight Loss

**DOI:** 10.1155/2018/5172091

**Published:** 2018-05-22

**Authors:** Alice S. Ryan

**Affiliations:** VA Maryland Health Care System, Research Service, Department of Medicine, Division of Gerontology and Geriatric Medicine, University of Maryland School of Medicine and Baltimore Geriatric Research Education and Clinical Center (GRECC), Baltimore, MD 21201, USA

## Abstract

The purpose of this study was to compare systemic inflammation in older women with a history of gestational diabetes (GDM) who developed impaired glucose tolerance (IGT) or type 2 diabetes (T2DM) to that in those with normal glucose tolerance (NGT) and to determine, in these women, the effect of weight loss (WL) induced by diet and exercise training on systemic inflammation and adipokine levels. This was a longitudinal clinical investigation of overweight/obese (BMI: 32 ± 1 kg/m^2^) women (59 ± 1 years) with a GDM history (*n* = 19) who had normal glucose tolerance (NGT, *n* = 7) or IGT/T2DM (*n* = 12). Women completed 6 months of weight loss induced by diet and exercise and underwent VO_2_max, body composition, blood draw, glucose tolerance testing, and 2-hour hyperinsulinemic-euglycemic clamps (40 mU·m^−2^·min^−1^). Glucose utilization (M) was 42% higher in the NGT group (*P* < 0.05). CRP was two-fold higher in the IGT/T2DM group than that in the NGT group (*P* < 0.01). Adiponectin levels were 59% higher in the NGT group than those in the IGT/T2DM group (*P* < 0.01). IL-6sR was higher in the NGT group (*P* < 0.01). The women lost body weight, body fat, visceral fat, and subcutaneous abdominal fat (*P* < 0.001). Fasting glucose (*P* < 0.05), fasting insulin, glucose, and insulin AUC decreased (all *P* < 0.005) after the intervention. M increased by 21% (*P* < 0.05). CRP (−16%) and TNFR1 (−6%) tended to decrease, whereas TNF*α*, IL-6, SAA, and adiponectin did not change in the group. In conclusion, older women with a history of GDM who have developed IGT or T2DM have higher CRP and reduced adiponectin levels despite similar BMI and total and abdominal obesity to those with NGT. Six months WL induced by diet and exercise improves body composition and increases insulin sensitivity without a significant modification of inflammatory markers and adiponectin levels.

## 1. Introduction

The global prevalence estimate of total diabetes in pregnancy (e.g., both known diabetes in pregnancy and previously undiagnosed diabetes in pregnancy) is nearly 17% [1]. Gestational diabetes mellitus (GDM) carries a long-term risk of developing type 2 diabetes for the mother with estimates reported as an overall relative risk of 6.0 [2] and a 7–12-fold greater lifetime risk for subsequent development of type 2 diabetes [3]. Our results indicate that postmenopausal women with prior GDM are more insulin resistant than postmenopausal women controls of similar age, adiposity, and fitness levels and are as insulin resistant as women with T2DM [4] further supporting conclusions that women with GDM are at risk for diabetes.

Inflammation is associated with increased risk for cardiovascular disease, and inflammatory markers are elevated in obesity and diabetes. Inflammatory markers are, for the most part, elevated during pregnancy in GDM women compared to those in non-GDM women [5]. Furthermore in a systematic meta-analysis, maternal adiponectin levels are significantly lower and TNF*α* and leptin are higher in GDM patients than those in controls [6]. It is, therefore, reasonable to hypothesize that inflammation may play a part in the heightened insulin resistance observed in older women with a history of GDM.

In a recent meta-analysis of approximately 30 randomized controlled trials of either diet, physical activity, or both, lifestyle interventions during pregnancy resulted in an 18% reduction in the risk of GDM, but a lifestyle intervention initiated at or after the 16th week of gestation did not reduce the risk of GDM [7]. Lifestyle programs may also be beneficial postpartum in women with a history of GDM, wherein an increase in physical activity can halve the risk of developing type 2 diabetes mellitus [8], and even a small amount of postpartum weight loss is associated with improved glucose metabolism [9]. In older women with a history of GDM, we reported that aerobic exercise with moderate weight loss reduces body weight and visceral and subcutaneous abdominal fat and improves insulin sensitivity [10]. Yet, the effects of lifestyle modification on adipokines were not investigated in older women with a history of GDM. However, in a broad context, modest weight loss alone or with exercise reduces inflammatory markers in overweight and obese adults [11–14].

Although our data suggest that older overweight and sedentary women with a history of GDM are insulin resistant, the role of inflammation in this context is unknown. The hypothesis tested was that inflammatory markers would be higher and adiponectin levels would be lower in women with a history of gestational diabetes who later developed impaired glucose tolerance (IGT) or type 2 diabetes than in women with a history of GDM who have normal glucose tolerance and that lifestyle modification would reduce inflammatory profiles. Therefore, the aim of this study was to compare systemic inflammation in older women with a history of GDM who developed IGT or type 2 diabetes to that in those with NGT and to determine, in these women, the effect of weight loss induced by diet and exercise training on systemic inflammation and adipokine levels.

## 2. Materials and Methods

### 2.1. Study Sample and Study Intervention

Women were recruited from the Baltimore metropolitan area for participation in this study. Each had a history of GDM between 5 and 32 years prior as confirmed by a physician or healthcare provider. Women were overweight and obese with a body mass index (BMI) between 26 and 38 kg/m^2^ and were 41–68 years of age. They were weight stable, defined as <2.0 kg weight change in past year, as well as sedentary, defined as performing aerobic exercise for <20 min twice per week. The University of Maryland Institutional Review Board approved the study. Each woman provided written informed consent. Subjects underwent a screening process, which included a medical history and physical exam, fasting blood chemistries, a 12-lead resting electrocardiogram, and graded exercise treadmill test. Exclusion criteria included hormone replacement therapy; smoking; cancer; or any indication of liver, renal or hematological disease, or other medical disorders.

Nineteen eligible women completed a 6-month weight loss program induced by diet and aerobic exercise. The effects of weight loss on body composition and insulin sensitivity were reported in a larger sample size (*n* = 25); however, the influence of glucose tolerance on inflammatory cytokines and adiponectin levels were not examined. All women attended weekly weight loss classes led by a registered dietitian for instruction in the American Heart Association (AHA) Step I [15] guidelines for weight loss. They were instructed to restrict their caloric intake by 250–350 kcal/d. Some women exercised on motorized treadmills and cycle ergometers 3 times/week, 45 min/session, for 6 months. Exercise intensity was prescribed as ~50–60% heart rate reserve and gradually progressed in duration and intensity to >60% VO_2_max for 45 minutes. The heart rate was monitored during exercise using heart rate monitors (Polar Electro Inc., Lake Success, NY).

### 2.2. Research Testing

Before and after 6 months of weight loss, subjects underwent research testing consisting of body composition measurements, maximal exercise tests, oral glucose tolerance tests, and blood draws. Height (cm) and weight (kg) were determined for BMI (kg/m^2^). Subjects underwent dual-energy X-ray absorptiometry (DXA) (Model DPX-L, Lunar Radiation Corp., Madison, WI) scan to measure fat mass, lean tissue mass, and bone mineral content (BMC) and to calculate fat-free mass (FFM = lean tissue + BMC). Women also had an L_4_-L_5_ abdominal computed tomography (CT) scan (PQ 6000 Scanner General Electric Hi-Light, Cleveland, Ohio) for determination of visceral (VAT) and subcutaneous abdominal adipose tissue (SAT) areas [10]. A continuous treadmill test protocol [16] was used to determine VO_2_max, whereby subjects met two of three following criteria including a plateau in oxygen uptake with an increased work load as evidenced by a difference in the oxygen uptake of <2 mL·kg^−1^·min^−1^, a respiratory exchange ratio of >1.10, or a maximal heart rate within 10 beats/min of the age-predicted maximal value.

All testing was performed in the morning after a 12-hr overnight fast. All subjects were weight stabilized (<1 kg) for at least two weeks prior to testing. Subjects underwent a 75-g, 2-h oral glucose tolerance test (OGTT) [17] with blood samples drawn every 30 min for measurement of plasma glucose and insulin levels. For two days prior to the glucose clamps, the RD provided the subjects with two days of an isocaloric diet (55–60% carbohydrates, 15–20% protein, and <30% fat). The number of calories given to each woman was estimated from the 7-day food records, and estimates of energy expenditure were based on the Harris-Benedict equation [18]. Peripheral tissue sensitivity to exogenous insulin was measured using the hyperinsulinemic-euglycemic clamp technique [19] with a priming and continuous infusion of insulin for two hours (240 pmol·m^−2^·min^1^, Humulin, Eli Lilly Co., Indianapolis, IN). The infusion of glucose and insulin was made through an intravenous catheter, and blood samples were drawn from a dorsal hand or wrist vein in which the hand was warmed to “arterialize” [20] the blood through the use of a warmed chamber. The amount of glucose infused after glucose space correction determined glucose utilization or M. One woman in the NGT group and five women in the IGT/T2DM did not undergo the glucose clamp because of venous access difficulty or scheduling conflict.

### 2.3. Blood Sample Analyses (Cytokines, Adiponectin, Glucose, and Insulin)

Blood samples were transferred into chilled tubes containing 1 mg of EDTA per cc of blood. The plasma was separated by centrifugation at 4°C for 15 min at 2000 ×g. Plasma IL-6, TNF*α*, their soluble receptors (sIL-6R, TNFR1), adiponectin, glucose, and insulin were measured in duplicate, and the average of the two values was used for data analyses. Duplicate samples that did not provide a coefficient of variation less than 15% were reanalyzed, and all values were averaged for data analyses. All cytokines and cytokine-soluble receptors were measured using Quantikine ELISA kits (R&D Systems, Minneapolis, MN). The inter- and intra-assay CVs were 6% for IL-6 and sIL-6R and 12% for TNF*α* and TNFR1. The CRP was measured in triplicate using an automated immunoanalyzer (Immulite, Diagnostics Products Corp., Los Angeles, CA) with inter- and intra-assay CVs of 7.5 and 4.4%, respectively. SAA levels were measured in triplicate with an ELISA kit according to the manufacturer's instructions (BioSource, Camarillo, CA, USA) with the intra-assay CV of 4%. Adiponectin was determined by RIA (Millipore, St. Charles, MO) with the inter- and intra-assay coefficient of variations of <10% at ED20, ED50, and ED80 concentrations of the standard range. Plasma insulin and leptin were measured by RIA (Millipore, St. Charles, MO) and glucose with the glucose oxidase method (2300 STAT Plus; YSI, Yellow Springs, OH).

## 3. Statistical Analyses

The statistical significance between glucose tolerance groups was determined by unpaired *t*-tests. Changes with the treatment were determined by paired *t*-test. Relationships between variables were determined by linear regression analyses and calculation of Pearson correlation coefficients. Data were analyzed using SPSS (PASW Statistics 18, SPSS Inc., Chicago, IL) and expressed as mean ± standard error of the mean (SEM). Statistical significance was set at the *P* < 0.05.

## 4. Results

### 4.1. Baseline Characteristics

**Figure 1:**
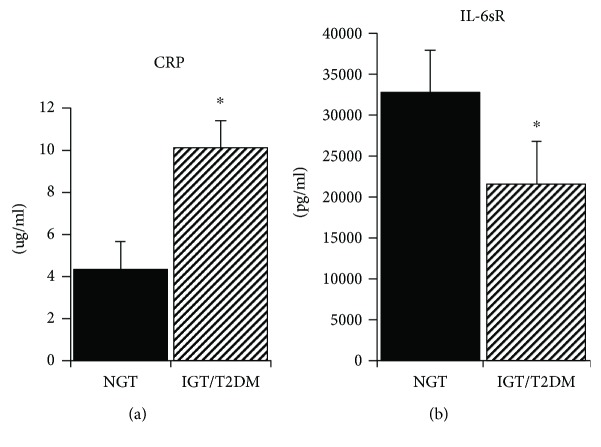
(a) CRP levels in NGT and IGT/T2DM groups. Values are mean ± SEM. Significantly different between groups, ^∗^*P* < 0.01. (b). IL-6sR levels in NGT and IGT/T2DM groups. Values are mean ± SEM. Significantly different between groups, ^∗^*P* < 0.01.

**Figure 2:**
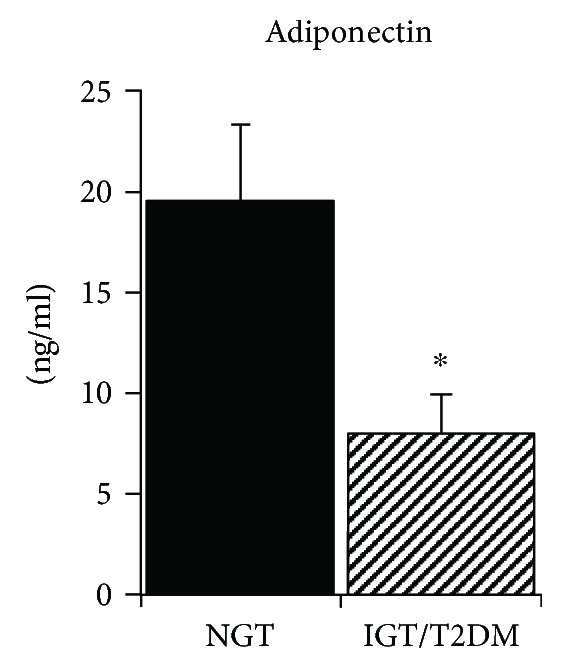
Adiponectin levels in NGT and IGT/T2DM groups. Values are mean ± SEM. Significantly different between groups, ^∗^*P* < 0.01.

Of the 19 women with a history of GDM, seven women had NGT and 12 women had IGT (*n* = 5) or T2DM (*n* = 7). Only one woman was on an oral agent for T2DM, and the remaining six were untreated. The NGT and IGT/T2DM groups did not differ in age, body weight, BMI, VO_2_max, percent body fat, fat mass, or FFM (Table 1). The visceral adipose tissue area was higher in the NGT group (*P* < 0.05), but the SAT was not different between groups. Fasting glucose and insulin levels were not significantly different between groups, but as expected, G_120_ and glucose AUC was higher in the IGT/T2DM group than that in the NGT group (*P* < 0.0001). Glucose utilization (M) was 42% higher in the NGT group (*P* < 0.01). CRP was over twofold higher in the IGT/T2DM group than that in the NGT group (*P* < 0.01, Figure 1(a)). Fasting plasma SAA, TNF*α*, TNFR1, and IL-6 did not differ between groups at baseline (Table 1). IL-6sR was higher in the NGT group (*P* < 0.01, Figure 1(b)). Adiponectin levels were 59% higher in the NGT group than those in the IGT/T2DM group (*P* < 0.01, Figure 2). Insulin AUC increased with age (*r* = 0.56, *P* < 0.05). VO_2_max (*r* = −0.47, *P* < 0.05) and M (*r* = −0.59, *P* < 0.05) decreased with age.

### 4.2. Relationships with Inflammation

Relationships between cytokines and clinical phenotypes indicate that IL-6sR was negatively associated with G_120_ (*r* = −0.62, *P* < 0.05) and glucose AUC (*r* = −0.72, *P* < 0.01) but not insulin AUC. The CRP was negatively associated with M (*r* = −0.56, *P* < 0.05). Adiponectin was negatively associated with fasting glucose (*r* = −0.50, *P* < 0.05), G_120_ (*r* = −0.65, *P* < 0.01), and fasting insulin (*r* = −0.59, *P* < 0.05) and positively with M (*r* = 0.63, *P* < 0.05). Of all the cytokines, only IL-6 increased with age (*r* = 0.52, *P* < 0.05). There were some relationships with total and abdominal adiposity such that the percent body fat was associated with higher CRP (*r* = 0.64, *P* < 0.01) and SAA (*r* = 0.49, *P* < 0.05). VAT predicted TNFR1 (*r* = 0.67, *P* < 0.01) and IL-6sR (*r* = 0.55, *P* < 0.05) and approached significance for SAA (*r* = 0.47, *P* = 0.08). SAT predicted SAA (*r* = 0.53, *P* < 0.05). Relationships between cytokines indicate that TNFR1 is related to IL-6sR (*r* = 0.68, *P* < 0.01) and CRP is related to SAA (*r* = 0.51, *P* < 0.05).

### 4.3. Effects of Interventions (Table 2)

In the total group or in the NGT and IGT/T2DM groups, there were significant losses of body weight (total group: −7.4 ± 1.0%, NGT: −7.1 ± 1.7%, IGT/T2DM: −7.6 ± 1.4%, all *P* < 0.005). There were also significant reductions in BMI, body fat, visceral fat, and subcutaneous abdominal fat (*P* = 0.01–<0.0001). There was a 7% increase in VO_2_max (l/min) in the total group (*P* < 0.05) and VO_2_max increased in both the NGT group and the IGT/T2DM group (*P* < 0.05). Fasting glucose (*P* < 0.05), fasting insulin (*P* < 0.005), and 120-min glucose (*P* < 0.01) decreased along with a 14 and 31% reduction in glucose AUC (*P* < 0.0001) and insulin AUC (*P* < 0.005), respectively, after WL treatments in the total group. Fasting, 120 min glucose, and glucose AUC did not significantly change but fasting insulin and insulin AUC decreased in the NGT group (*P* < 0.05). Fasting, 120-min glucose, glucose, and insulin AUC decreased in the IGT/T2DM group (P's < 0.05). M increased by 21% (*P* = 0.05) in the total group and increased in the NGT group (*P* < 0.05). Plasma leptin decreased by 31% (*P* < 0.01) in the total group and decreased in the IGT/T2DM group (*P* < 0.0001). There were no significant changes in CRP, TNF*α*, TNFR1, IL-6, SAA, and adiponectin after WL in the total group or NGT and IGT/T2DM groups.

## 5. Discussion

In studies that have examined inflammatory markers *during* pregnancy, results are somewhat conflicting. Women with GDM (*n* = 200) had higher TNF*α* but not CRP in the first trimester than 800 unaffected women [21], whereas higher CRP levels were observed in another case-control study of GDM women (*n* = 36) [22]. Adiponectin levels are reported as lower in 30 GDM women compared to the same number of healthy pregnant women [23]. In another study, CRP and TNF*α* levels were higher in GDM than those in NGT pregnant women, but differences disappeared when adjusting for age, family history of T2DM, and previous GDM history and prepregnancy BMI [24]. It is possible that differences in the timing of measurements (trimester), adjustment for confounders, sample sizes, and ethnicity may help explain these disparate results. However, a systematic review and meta-analysis concluded from 27 trials that maternal TNF*α* and leptin levels are higher in GDM women than those in controls, and adiponectin concentrations are lower [6], which would support that some inflammatory markers are altered during pregnancy.

There is limited information regarding inflammatory markers in women with a prior *history* of GDM. In young women who had GDM about seven years prior to enrolment, soluble TNF*α*R2 and IL-6 levels were higher than those in young women without a history of GDM [25]. In another cross-sectional study of predictors of inflammatory markers in young women ~three years after pregnancy complicated by GDM, higher waist circumference was significantly associated with higher CRP, leptin, and resistin and negatively with adiponectin [26]. Although the majority of women (~70%) did not meet recommended weekly levels of physical activity, participation in physical activity lowered cardiometabolic risk in women with prior GDM. In young women with GDM, the skeletal muscle TNF*α* gene expression was not only 5–6 times higher than that in women with NGT during late pregnancy but also remained threefold higher than that in NGT women at one year postpartum follow-up visit [27]. Further, circulating TNF*α* did not change postpartum in the GDM women compared to a significant decline in the NGT women suggesting that inflammation persists after GDM.

The current study is unique in its examination of older women with a history of GDM. Although our study did not compare older women with and without a history of GDM, results from our previous study of postmenopausal women (*n* = 58) without a history of GDM [13], levels of CRP, TNF*α*, TNFR1, IL-6, and IL-6sR do not appear different from those of the women in the current study with a history of GDM. In the current study, where all women had a history of GDM, those who developed IGT or type 2 diabetes had higher CRP levels, but TNF*α*, TNFR1, IL-6, and SAA were not significantly different between groups. We have shown that the total body fat mass and subcutaneous abdominal fat are associated with insulin resistance in women with a history of GDM [4] and now add that increased CRP is related to reduced insulin sensitivity. We are also unaware of any studies that have examined adiponectin concentrations in older women with a history of GDM. Our results indicate that the IGT/T2DM group had lower adiponectin levels than the NGT women with a history of GDM. Further, higher adiponectin levels were associated with insulin sensitivity by the glucose clamp in this group of women with a history of GDM, which is similar to our previous study in women without a history of GDM [28]. Thus, inflammation as indicated by high CRP and low adiponectin may alter the risk for the development of type 2 diabetes after pregnancy complicated by GDM.

In overweight and obese postmenopausal women without a history of GDM, we have previously demonstrated a reduction in CRP [11, 13], IL-6 and sTNFR1 [13], and SAA [11, 12, 29] levels with either weight loss or aerobic training plus weight loss. We also previously reported a decrease in plasma leptin after weight loss alone and with aerobic exercise in women with a history of GDM [10], which is consistent with the reduction in plasma leptin in the current study. High plasma leptin predicted low insulin sensitivity in a young group of women with recent GDM, suggesting that leptin signaling may be more important than low-grade inflammation as a contributor to type 2 diabetes [30]. Yet, changes in cytokines were not significant during the same interventions in the current study. Although these studies were conducted with very similar designs and methods for the interventions and had similar reductions in body weight, sample sizes were two to three times larger in our earlier work so it is possible that we did not have a sufficient sample size to detect a change with the interventions. In addition, we were unable to distinguish differences in changes in cytokines between women who developed IGT and those who developed type 2 diabetes. It is possible that the magnitude of the effect would be vary between these groups.

The literature is lacking in terms of long-term follow-up of middle-aged and older women with prior history of GDM and the identification of mechanisms for the persistent insulin resistance. It is also possible that different inflammatory sources such as adipose tissue or endothelial cells could contribute to the inflammation observed after GDM. Future work could be directed to investigating these gaps while the results herein provide support for higher inflammation and insulin resistance in older women with a history of GDM.

## 6. Conclusions

Results of this study indicate that older women with a history of GDM who had developed IGT or T2DM have higher CRP and lower adiponectin levels than women who had NGT despite similar fitness and adiposity, which was related to insulin resistance and insulin sensitivity, respectively, by the glucose clamp. Yet, changes in inflammation were modest with a lifestyle intervention in this group of older women. Thus, in older women with a history of GDM, inflammation appears to play a role in the insulin resistance, although other factors may be more influential for the metabolic improvements observed with weight loss and exercise.

## Figures and Tables

**Table 1:** Baseline body composition, glucose metabolism, and inflammation.

	NGT (*n* = 7)	IGT/T2DM (*n* = 12)
Age (year)	50 (2)	52 (2)
Weight (kg)	85.9 (4.4)	85.1 (3.0)
Body mass index (kg/m^2^)	32.2 (1.5)	32.0 (0.9)
Percent body fat	46.1 (1.7)	45.4 (1.4)
Fat mass (kg)	39.5 (2.7)	38.3 (2.5)
Fat-free mass (kg)	45.9 (2.2)	47.3 (2.6)
VO_2_max (L/min)	1.93 (0.10)	1.72 (0.09)
Visceral fat (cm^2^)	150.2 (22.9)	127.7 (10.9)^a^
Subcutaneous abdominal fat (cm^2^)	457.3 (38.2)	484.9 (42.6)
Fasting glucose (mmol/L)	5.2 (0.1)	6.3 (0.4)
Glucose at 120 min (mmol/L)	5.9 (0.5)	11.1 (0.6)^c^
Fasting insulin (pmol/L)	76 (13)	107 (15)
G_AUC_ (mmol/L/120 min)	862 (25)	1231 (57)^c^
I_AUC_ (pmol/L/120 min)	55,700 (9209)	56,794 (9954)
Glucose utilization (*μ*mol/kg/min)	31.9 (4.6)	17.7 (2.4)^b^
Glucose utilization (*μ*mol/kg_FFM_/min)	57.9 (7.2)	63.8 (3.1)^a^
TNF*α* (pg/mL)	2.93 (1.92)	1.51 (0.36)
TNFR1(*μ*g/mL)	808.9 (84.7)	744.9 (48.5)
IL-6 (pg/mL)	2.08 (0.30)	2.38 (0.52)
SAA (*μ*g/mL)	43.1 (9.65)	64.7 (37.1)

Values are mean (SEM). Baseline differences tested between groups: ^a^*P* < 0.05, ^b^*P* < 0.01, and ^c^*P* < 0.001. NGT: normal glucose tolerance; IGT/T2DM: impaired glucose tolerance/type 2 diabetes mellitus; VO_2_max: maximum oxygen uptake; G_AUC_: glucose area under the curve; I_AUC_: insulin area under the curve.

**Table 2:** Effects of the interventions on body composition, glucose metabolism, and inflammation.

	NGT/IGT/T2DM (*n* = 19)		NGT (*n* = 7)		IGT/T2DM (*n* = 12)	
	Before	After	Before	After	Before	After
Age (yr)	52 ± 2		50 ± 2		52 ± 2	
Weight (kg)	85.4 ± 2.4	79.2 ± 2.6^d^	85.9 ± 4.4	79.6 ± 3.7^b^	85.1 ± 3.0	78.9 ± 3.6^d^
Body mass index (kg/m^2^)	32.0 ± 0.7	29.9 ± 0.9^d^	32.2 ± 1.5	29.9 ± 1.4^b^	31.9 ± 0.9	29.9 ± 1.2^b^
Percent body fat	45.6 ± 1.1	42.2 ± 1.5^d^	46.1 ± 1.7	42.2 ± 2.1^c^	45.4 ± 1.4	42.1 ± 2.1^b^
Fat mass (kg)	38.7 ± 1.8	33.9 ± 2.1^d^	39.5 ± 2.7	33.6 ± 2.8^c^	38.3 ± 2.5	34.0 ± 3.0^c^
Fat-free mass (kg)	46.8 ± 1.8	44.9 ± 1.1	45.9 ± 2.2	45.4 ± 1.9	47.3 ± 2.6	44.7 ± 1.3
VO_2_max (L/min)	1.80 ± 0.07	1.92 ± 0.07^c^	1.92 ± 0.09	1.91 ± 0.09	1.72 ± 0.09	1.92 ± 0.09^c^
Waist circumference (cm)	93.4 ± 1.9	88.1 ± 2.1^d^	92.8 ± 4.4	88.6 ± 4.1^b^	93.7 ± 1.6	87.8 ± 2.5^c^
Hip circumference (cm)	112.2 ± 1.5	105.7 ± 1.6^d^	114.8 ± 2.3	106.8 ± 1.8 ^a^	110.5 ± 1.9	104.9 ± 2.5^c^
Visceral fat (cm^2^)	137.5 ± 11.6	110.0 ± 10.1^d^	150.2 ± 22.9	121.8 ± 20.1^a^	127.7 ± 10.9	100.9 ± 8.9^c^
Subcutaneous abdominal fat (cm^2^)	472.8 ± 28.5	414.5 ± 31.2^d^	457.3 ± 38.2	399.1 ± 40.4^a^	484.9 ± 42.6	426.5 ± 47.4^c^
Sagittal diameter (cm)	25.9 ± 0.7	24.8 ± 0.7^d^	25.3 ± 1.1	23.7 ± 1.0^a^	26.3 ± 0.9	24.6 ± 1.0^c^
Fasting glucose (mmol/L)	5.9 ± 0.3	5.6 ± 0.2^a^	5.2 ± 0.1	5.1 ± 0.2	6.3 ± 0.4	5.9 ± 0.3 ^a^
Glucose at 120 min (mmol/L)	9.4 ± 0.7	7.9 ± 0.6^b^	6.2 ± 0.3	5.9 ± 0.5	11.1 ± 0.6	9.0 ± 0.6 ^a^
Fasting insulin (pmol/L)	93 ± 11	68 ± 8^c^	76 ± 13	68 ± 11	107 ± 15	67 ± 11^c^
G_AUC_ (mmol/L/120 min)	1092 ± 58	941 ± 46^d^	862 ± 25	779 ± 51	1231 ± 57	1037 ± 45^d^
I_AUC_ (pmol/L/120 min)	56,289 ± 6558	38,821 ± 4868^c^	55,700 ± 9209	45,816 ± 8108^a^	56,794 ± 9954	32,825 ± 5300^a^
Glucose utilization (*μ*mol/kg/min)	23.9 ± 3.3	28.9 ± 3.3^a^	32.0 ± 4.6	37.5 ± 3.9^a^	17.1 ± 2.9	21.4 ± 3.2
Glucose utilization (*μ*mol/kg_FFM_/min)	43.3 ± 5.9	49.9 ± 5.5	57.9 ± 7.2	64.8 ± 6.2	30.8 ± 5.9	37.1 ± 5.2
CRP (*μ*g/mL)	7.24 ± 1.11	7.10 ± 1.18	4.87 ± 1.26	4.40 ± 1.25	9.08 ± 1.49	9.10 ± 1.60
TNF*α* (pg/mL)	2.13 ± 0.85	2.08 ± 0.51	2.93 ± 1.92	1.91 ± 0.98	1.51 ± 0.36	2.21 ± 0.54
TNFR1 (*μ*g/mL)	772.9 ± 45.1	724.3 ± 42.5	808.9 ± 84.7	675.6 ± 73.5	744.9 ± 48.5	762.1 ± 49.7
IL-6 (pg/mL)	2.25 ± 0.31	2.09 ± 0.24	2.08 ± 0.30	2.01 ± 0.42	2.38 ± 0.52	2.15 ± 0.29
IL-6sR (pg/mL)	26,301 ± 2144	27,521 ± 2169	32,994 ± 3406	31,612 ± 4044	21,839 ± 1533	24,793 ± 2142
Adiponectin (ng/mL)	12.6 ± 2.4	12.8 ± 2.6	19.6 ± 3.8	20.1 ± 5.0	8.34 ± 2.2	8.42 ± 2.1
Leptin (pmol/L)	26.0 ± 2.3	19.9 ± 2.1^b^	23.7 ± 4.0	20.3 ± 3.5	27.8 ± 2.6	19.7 ± 2.7^d^
SAA (*μ*g/mL)	55.4 ± 21.2	29.5 ± 4.6	43.1 ± 9.6	28.4 ± 7.9	64.7 ± 37.1	30.3 ± 5.9

Values are mean (SEM). Significantly different before and after the intervention: ^a^*P* < .05, ^b^*P* < .01, ^c^*P* < 0.005, and ^d^*P* < 0.001. VO_2_max: maximum oxygen uptake; G_AUC_: glucose area under the curve; I_AUC_: insulin area under the curve.

## Data Availability

The research data used to support the findings of this study are available from the corresponding author upon request, for researchers who meet the criteria for access to confidential data.
